# Prognostic Factors and Nomogram for Choroid Plexus Tumors: A Population-Based Retrospective Surveillance, Epidemiology, and End Results Database Analysis

**DOI:** 10.3390/cancers16030610

**Published:** 2024-01-31

**Authors:** Abhishek S. Bhutada, Srijan Adhikari, Joshua A. Cuoco, Alexander In, Cara M. Rogers, John A. Jane, Eric A. Marvin

**Affiliations:** 1 Virginia Tech Carilion School of Medicine, 2 Riverside Circle, Roanoke, VA 24016, USA; sadhikari@carilionclinic.org (S.A.); jacuoco@carilionclinic.org (J.A.C.); alexanderin@vt.edu (A.I.); cmrogers@carilionclinic.org (C.M.R.); jajane@carilionclinic.org (J.A.J.J.); eamarvin@carilionclinic.org (E.A.M.); 2 Department of Neurosurgery, Carilion Clinic, 1906 Belleview Avenue, Roanoke, VA 24014, USA; 3 School of Neuroscience, Virginia Polytechnic Institute and State University, Blacksburg, VA 24061, USA

**Keywords:** choroid plexus tumors, prognostic factors, survival analysis, central nervous system, nomogram

## Abstract

**Simple Summary:**

Given the rarity of choroid plexus tumors (CPTs), there is a need for large cohort analyses to provide a better understanding of prognostication and optimal diagnosis. Using the National Cancer Institute’s Surveillance, Epidemiology, and End Result (NCI SEER) database, here we present one of the largest retrospective cohort analyses studying patients with CPT. This study provides a better understanding of prognostication and optimal treatment, especially the role that neoadjuvant therapies play.

**Abstract:**

*Background:* Choroid plexus tumors (CPTs) are rare neoplasms found in the central nervous system, comprising 1% of all brain tumors. These tumors include choroid plexus papilloma (CPP), atypical choroid plexus papilloma (aCPP), and choroid plexus carcinoma (CPC). Although gross total resection for choroid plexus papillomas (CPPs) is associated with long-term survival, there is a scarcity of prospective data concerning the role and sequence of neoadjuvant therapy in treating aCPP and CPC. *Methods:* From the years 2000 to 2019, 679 patients with CPT were identified from the Surveillance, Epidemiology, and End Result (SEER) database. Among these patients, 456 patients had CPP, 75 patients had aCPP, and 142 patients had CPC. Univariate and multivariable Cox proportional hazard models were run to identify variables that had a significant impact on the primary endpoint of overall survival (OS). A predictive nomogram was built for patients with CPC to predict 5-year and 10-year survival probability. *Results:* Histology was a significant predictor of OS, with 5-year OS rates of 90, 79, and 61% for CPP, aCPP, and CPC, respectively. Older age and African American race were prognostic for worse OS for patients with CPP. Older age was also associated with reduced OS for patients with aCPP. American Indian/Alaskan Native race was linked to poorer OS for patients with CPC. Overall, treatment with gross total resection or subtotal resection had no difference in OS in patients with CPP or aCPP. Meanwhile, in patients with CPC, gross total resection (GTR) was associated with significantly better OS than subtotal resection (STR) only. However, there is no difference in OS between patients that receive GTR and patients that receive STR with adjuvant therapy. The nomogram for CPC considers types of treatments received. It demonstrates acceptable accuracy in estimating survival probability at 5-year and 10-year intervals, with a C-index of 0.608 (95% CI of 0.446 to 0.77). *Conclusions:* This is the largest study on CPT to date and highlights the optimal treatment strategies for these rare tumors. Overall, there is no difference in OS with GTR vs. STR in CPP or aCPP. Furthermore, OS is equivalent for CPC with GTR and STR plus adjuvant therapy.

## 1. Introduction

Although choroid plexus tumors (CPTs) are rare neoplasms, they account for 12–20% of brain tumors in infants within their first year of life [1,2,3,4]. The choroid plexus is a highly vascularized stroma surrounded by neuroectoderm-derived cells anatomically located on the ventricle walls [5,6]. Physiologically, the role of the choroid plexus is to produce cerebrospinal fluid (CSF), maintain the blood–CSF barrier, guide neural tissue development, and house stem cells [5].

The World Health Organization classifies CPTs into three types: choroid plexus papillomas (CPCs), atypical choroid plexus papillomas (aCPPs), and choroid plexus carcinoma (CPC) [7]. The majority of cases are located in the ventricles; however, some reports have shown this neoplasm located outside of the ventricles, particularly in specific age groups [8]. For example, CPPs seem to occur more often in the third and fourth ventricles compared to CPCs [1,9]. Since the fourth ventricle contains a large and more prominent choroid plexus compared to the lateral ventricles in adults, the likelihood of tumor development is higher in the fourth ventricle [9]. In general, CPPs have a more favorable clinical course after gross total resection. Still, recurrences spreading along the neuraxis have been reported [10,11]. Contrarily, CPCs are characterized by high mitotic activity, dense cellularity, and frequent brain invasion [12]. Craniospinal dissemination in CPC has been shown to occur in 12–30% of cases [9,11,13].

CPPs tend to have favorable prognosis with reported one- and five-year survival rates of 90 and 81%, respectively, along with good long-term clinical outcomes with complete surgical resection [9,11]. Unlike CPPs, CPCs are aggressive malignant tumors with poor prognosis one- and five-year survival rates of 71 and 41%, respectively [9]. Those patients who do survive most often present with cognitive and developmental deficits [9]. Recurrence and craniospinal disseminations are more common in CPCs than CPPs, with rates ranging from 12 to 30% [11,13]. The survival rates for aCPPs are less well defined due to the limited studies surrounding this tumor. One study reported a five-year survival rate of 83% [11]. Resection of the tumor is the current approach for both CPPs and CPCs, with others including chemotherapy and/or radiation therapy [9,11]. Further data are needed regarding the efficacy of chemotherapy and radiation as there are reported cases demonstrating the benefits of radiation with and without resection [14,15].

Although the literature is sparse, there are clinical trials and preclinical studies with new therapies and possible treatment options, including new chemotherapy, immunotherapy drugs, and other targeted therapies. Martin et al., in 2023, used a genetic mouse model where they conducted a high-throughput screen on a human patient-derived CPC cell line and identified promising molecular targets that have the potential to treat CPC [16]. Moreover, others like Schell et al. (2001) demonstrated the use of adoptive T-cell transfer as immunotherapy against CPTs [17]. Determining the optimal therapy for CPC is challenging due to limited prospective data on the role and sequence of neoadjuvant therapy.

The National Cancer Institute’s Surveillance, Epidemiology, and End Result (NCI SEER) database compiles data on cancer from population-based registries, covering around 28% of the US population. This extensive dataset offers a valuable chance to study CPTs on a larger scale, examining clinical characteristics, treatments, and outcomes. Our study aims to classify prognostic features, assess therapy benefits, and highlight recent advancements in treating this rare group of tumors.

## 2. Materials and Methods

This study complies with the NCI SEER limited-use data end user agreement. All data used in this study are publicly available. Therefore, this study did not require any approval from an institutional review board.

### 2.1. Study Design and Cohort Selection

This retrospective cohort study examines patient data collected from the years 2000 to 2019 stored in the NCI SEER 18 database. All patients with choroid plexus tumors (CPTs) were identified using the International Classification of Diseases for Oncology, version 3 (ICD-O-3) morphology codes 9390/0, 9390/1, and 9390/3. The primary endpoint used for this study was overall survival (OS), defined as the time from diagnosis until the time of death, as reported by SEER. The SEER database provides information on cause of death; therefore, all individuals who die of causes other than the specific cancer diagnosis were treated as censored observations and excluded from the analysis. The prognostic factors that were evaluated included patient sex, age at diagnosis, race, primary tumor site, tumor size, treatments used, and extent of surgery. The treatments reported in the SEER database include surgery, radiation, and chemotherapy. Each of these treatment types were examined individually to assess their impact on patient survival. Furthermore, patients received varying combinations of these treatments. An analysis of the impact on different combination treatments was also examined.

### 2.2. Statistical Analyses

All statistical analyses were performed by R package (version 4.2.0, 2022; University of Auckland, Auckland, New Zealand) using the “survival”, “survminer”, and “rms” packages. Survival rate based on histology was calculated across all CPTs. Subsequent analyses looked at survival for patients within each specific tumor type based on histology: CPP, aCPP, and CPC. First, a univariate Cox proportional hazard model using the Breslow method for ties was performed to identify the factors that had a significant impact on OS. Based on the log-rank test, those factors that were found to have high significance (*p* < 0.05) were then included in multivariable analyses to test if significance was maintained. The Kaplan–Meier method was employed to visualize survival curves of each of the identified factors that maintained high significance after multivariable analyses. Finally, a nomogram for patients with CPC was created to predict survival probability.

## 3. Results

### 3.1. Overall Cohort Survival Statistics

**Figure 1:**
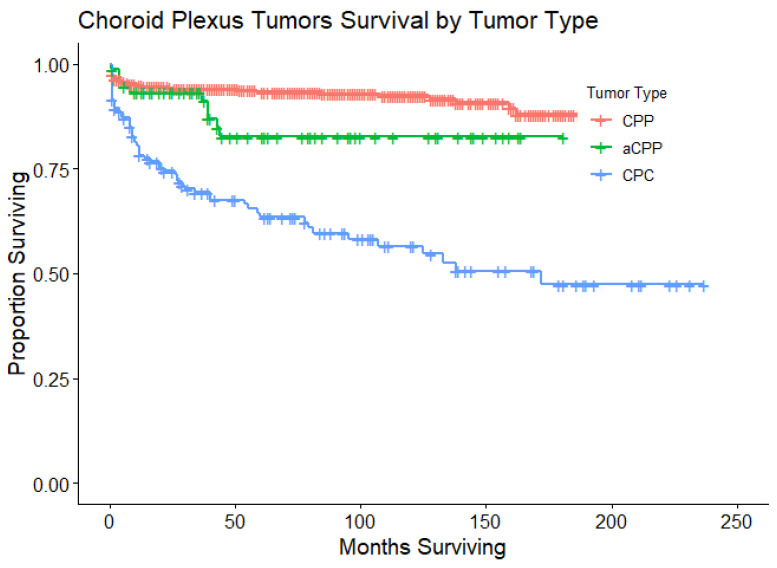
Kaplan–Meier curves that illustrate OS for patients with CPTs. This survival plot stratifies OS for patients by type of CPT: CPP (n = 462), aCPP (n = 75), and CPC (n = 142). Only patients with CPC had a statistically significant lower OS when compared to patients with CPP (*p* < 0.001). The median OS was not yet reached for patients with CPP or aCPP, while the median OS was 172 months for patients with CPC (95% CI 95 months to not yet reached).

A total of 679 patients with CPT were included in the present study. There were 462 patients with CPP (68%), 75 patients with aCPP (11%), and 142 patients with CPC (21%). Patients with CPP had a 1-year, 2-year, 5-year, and 10-year survival rate of 95%, 94%, 93%, and 88%, respectively. Patients with aCPP had a 1-year, 2-year, 5-year, and 10-year survival rate of 94%, 94%, 83%, and 83%, respectively. Patients with CPC had a 1-year, 2-year, 5-year, and 10-year survival rate of 80%, 76%, 65%, and 56%, respectively. As expected, the OS for patients with CPC was significantly worse than patients with CPP (*p* < 0.001). The OS for patients with aCPP showed no difference when compared to patients with CPP (*p* = 0.11). A Kaplan–Meier curve was created to illustrate the survival curves for each of these cohorts (Figure 1). For each tumor, the patient clinical demographics and treatments received are listed in Table 1. Additionally, for each tumor, the results from the univariate Cox proportional hazard ratio are listed in Table 2.

### 3.2. Choroid Plexus Papilloma

There were 462 patients with CPP, which accounted for 70% of all CPTs within the years of 2000 to 2019. Patients with CPP had a median OS that has yet to be reached with 35 patients that expired. Patient’s age and race were the statistically significant clinical characteristics to impact OS in patients with CPP. More specifically, ages ≥ 19 and African American race were associated with worse outcomes (Table 2).

There were 94 patients with CPP that did not receive any treatment. These patients were excluded from the analysis to focus on which treatments had a significant impact on OS. Patients received either gross total resection (273 patients; 59%), subtotal resection (172 patients; 37%), radiation (6 patients; 1%), gross total resection with chemotherapy (1 patient; <1%), gross total resection with radiation (3 patients; 1%), subtotal resection with chemotherapy (2 patients; <1%), subtotal resection with radiation (4 patients; 1%), or subtotal resection with radiation and chemotherapy (1 patient; <1%) (Table 1). There was no statistically significant difference in OS between patients that received gross total resection and patients who received subtotal resection (*p* = 0.22). Interestingly, the patients who received radiation or subtotal resection with radiation and chemotherapy had a statistically significant poorer OS compared to patients who received gross total resection (*p* < 0.001). Please refer to Table 2 for further details on hazard ratios and confidence intervals.

**Figure 2:**
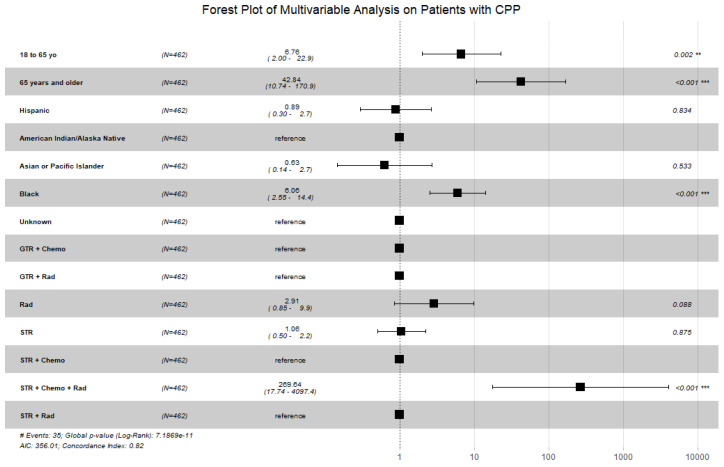
Forest plot displaying the multivariable Cox proportional hazard model for patients with CPP looking at patient age, patient race, and treatments received. Patients aged 18 years or older, African American race, and receipt of subtotal resection with radiation and chemotherapy remained statistically significant. (Abbreviations: GTR = gross total resection; STR = subtotal resection; Chemo = chemotherapy; Rad = radiation; # = number; ** = *p* < 0.01; *** = *p* < 0.001).

A multivariable Cox proportional hazard model was performed with patient age, race, and treatment since they were the factors that had a significant impact (*p* < 0.05) on OS. A forest plot was generated to display the results from this multivariable analysis (Figure 2). Patients aged 19 to 64 years old, patients aged 65 years or older, African American race, and receipt of subtotal resection with radiation and chemotherapy remained statistically significant.

### 3.3. Atypical Choroid Plexus Papilloma

There were 79 patients with aCPP, which accounted for 11% of all CPTs within the years of 2000 to 2019 with a median OS that has not yet been reached. Ten patients with aCPP died during this time period. Patients 65 years or older showed statistically significant poorer OS. All other prognostic factors did not show any statistical significance in terms of OS in the univariate analysis (Table 2). Multivariable analysis was not performed for this cohort given that there was only one statistically significant prognostic factor that was identified.

### 3.4. Choroid Plexus Carcinoma

There were 142 patients with CPC, which accounted for 19% of all CPTs within the years of 2000 to 2019. These patients had a median OS of 138 months (95% CI 82 months to not yet reached), with 61 patients that died. Patients that were American Indian/Alaskan Native had poorer OS compared to patients that were white. There were no other patient characteristics that had a statistically significant impact on OS in the univariate Cox proportional hazard model (Table 2).

There were nine patients with CPC who received no treatment. Similar to the analysis performed on patients with CPP, these patients who received no treatment were excluded to identify which treatment modalities had a significant impact on OS. Patients who received gross total resection were set as the reference group in the analysis. Patients received either gross total resection (30 patients; 21%), subtotal resection (8 patients; 6%), chemotherapy (4 patients; 3%), radiation (1 patient; 1%), gross total resection with chemotherapy (39 patients; 27%), gross total resection with radiation (12 patients; 8%), gross total resection with chemotherapy and radiation therapy (11 patients; 8%), subtotal resection with chemotherapy (27 patients; 19%), subtotal resection with radiation (4 patients; 3%), or subtotal resection with radiation and chemotherapy (6 patients; 4%) (Table 1). Patients who received only a subtotal resection had a statistically poorer OS when compared to patients who received gross total resection (*p* < 0.05). However, patients who received subtotal resection with adjuvant therapy (chemotherapy, radiation therapy, or both) had no difference in OS when compared to patients who received gross total resection (*p* > 0.05). This means that there is a survival benefit to utilizing neoadjuvant therapy in patients that receive subtotal resection. Please refer to Table 2 for further details on hazard ratios, confidence intervals, and respective *p*-values.

**Figure 3:**
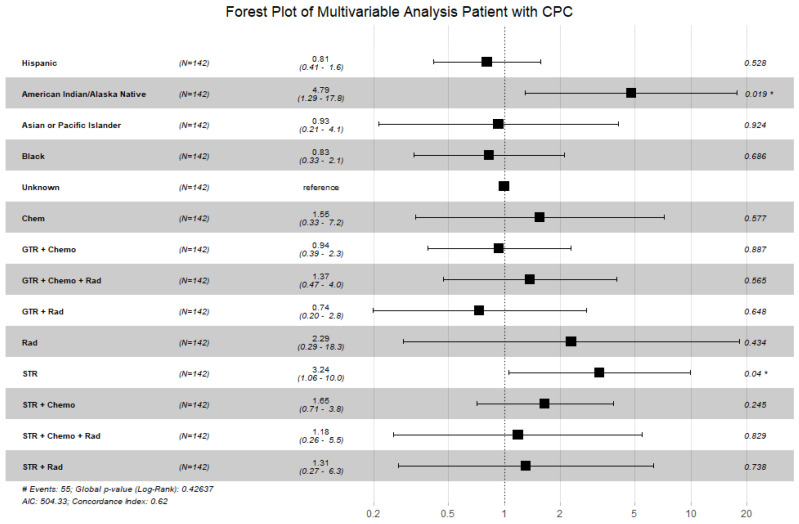
Forest plot displaying the multivariable Cox proportional hazard model for patients with CPC looking at race and treatments received. Patients of American Indian/Alaska Native race and patients that received subtotal resection remained statistically significant. Of note, the global *p*-value was 0.43; however, individual *p*-values for American Indian/Alaska Native race and subtotal resection were <0.05. (Abbreviations: GTR = gross total resection; STR = subtotal resection; Chemo = chemotherapy; Rad = radiation; # = number; * = *p* < 0.05).

A multivariable analysis was performed to see if patient race and treatment remain significant. A forest plot was generated to visualize these results (Figure 3). Patients of American Indian/Alaskan Native race and patients who received subtotal resection had poorer OS in the multivariable analysis. Of note, the global *p*-value was greater than 0.05 for this test.

### 3.5. Gross Total Resection Versus Subtotal Resection

**Figure 4:**
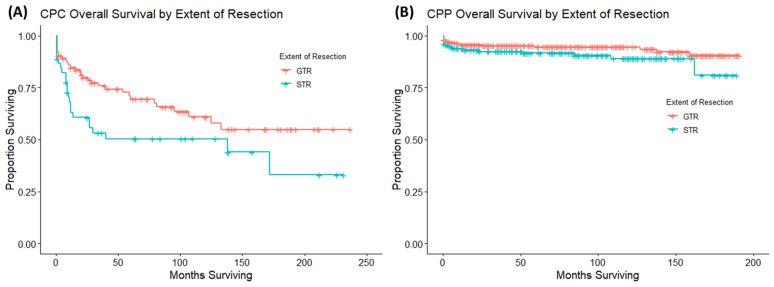
These are Kaplan–Meier curves looking at the extent of surgery on OS in patients with CPC (**A**) and patients with CPP (**B**). Receipt of gross total resection improved OS compared to patients that received subtotal resection in patients with CPC. Meanwhile, the extent of resection had no impact on OS in patients with CPP.

A subsequent analysis was conducted on patients with CPC and CPP to compare OS in patients who received either gross total resection or subtotal resection. Across patients with CPC, there were 92 patients who received gross total resection and 45 patients who received subtotal resection. Patients who received gross total resection had a statistically significant better OS than those patients who received subtotal resection. A Kaplan–Meier (KM) plot was built to visualize the survival curves of each group (Figure 4A). Across patients with CPP, there were 277 patients who received gross total resection and 179 patients who received subtotal resection. There was no significant difference in OS between the two groups using a univariate Cox proportional hazard model. A KM plot was built to visualize the survival curves of each group (Figure 4B).

### 3.6. Nomogram

**Figure 5:**
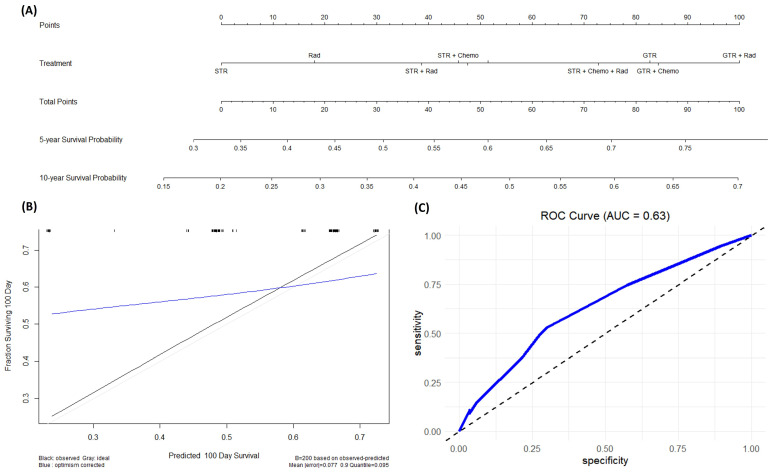
(**A**) This is a nomogram for patients with CPC looking at the effects of different treatments on survival probability. (**B**) This is a calibration plot that predicts the ability of this nomogram to correctly predict survival probability across the first 100 days from diagnosis. The black line represents the actual data, while the blue line represents the predicted data. This calibration plot has a mean error of 0.077 (**C**) This is an ROC plot evaluating the specificity and sensitivity of our model.

A nomogram was built on patients with CPC. An internal verification of both prognostic nomograms was calculated using the entire dataset and confirmed using bootstrapping verification. The nomogram built for patients with CPC also demonstrated acceptable accuracy in estimating the survival probability at 5-year and 10-year intervals with a C-index of 0.608 with a 95% CI of 0.446 to 0.77 (Figure 5).

## 4. Discussion

This study is one of the largest retrospective cohort analyses looking at patients with CPTs. As expected, CPCs had significantly worse OS compared to CPP and aCPP. There have only been two studies that utilized the SEER database to analyze CPTs; however, both studies consisted of a smaller sample size than the current study. Cannon et al., in 2015, utilized SEER data to not only evaluate overall survival but also the cause-specific survival of CPTs with a total of 349 CPT patients from 1978 to 2009 [18]. As another study with overlapping data, Lam et al. (2013) utilized SEER data to evaluate the overall survival of pediatric patients with CPT with a total of 168 CPT patients from 1978 to 2008 [19]. One of the major limitations of both studies was that these studies lack information on the use of chemotherapy due to the fact that at the time of their study, the SEER database did not contain this information. With the accumulation of more data regarding CPTs throughout the past decade, our study is currently the most up to date report of the prognostic factors and survival rate of this rare tumor.

### 4.1. Choroid Plexus Papilloma

There was a broader age distribution across patients with CPP, which resulted in an older mean age at diagnosis, consistent with older reports. Our study shows that patients with CPP 19 years or older have poorer OS. Similar findings were seen by Cannon et al. in 2015 [18]. One reason for this could be the fact that in adults, CPTs are found primarily in the third or fourth ventricle, whereas in pediatric patients, CPTs are found primarily in the lateral ventricles [19].

In our study, we found that the non-Hispanic black population had poorer OS compared to non-Hispanic white. To our knowledge, no other studies have shown this finding specific to CPP. However, multiple studies related to brain tumors in pediatric and adult patients have shown poorer OS in non-Hispanic black populations when compared to non-Hispanic white populations. Minority populations in the United States experience higher mortality rates from various cancers due to low socioeconomic status and limited healthcare access. Studies focusing on brain cancer disparities have shown that black patients, compared to white patients, are less likely to be treated by high-volume providers and experience increased complications after brain surgery, leading to potentially worse outcomes [20,21,22]. Differences in brain tumor survival among racial and ethnic groups could also be associated with variations in tumor biology, though the clinical significance of this remains not fully understood [22]. Additionally, racial and ethnic disparities in drug metabolism and other host-response factors may contribute to further variations in outcomes [23]. Addressing these issues is crucial to achieve more equitable cancer outcomes.

Based on our cohort, most patients with CPP were treated primarily with either gross total resection or subtotal resection. There are conflicting data on prior studies of whether or not gross total resection or subtotal resection improve OS in patients with CPP. Our study shows that there is no significant difference between patients that receive gross total resection versus those that receive subtotal resection. Due to the limited follow-up of CPP patients in this study, it cannot be definitively ruled out that gross total resection might offer a long-term overall survival benefit. The findings from a series of CPP cases at the Mayo Clinic indicated that subtotal resection is associated with an increased local failure rate of around 50%, but it may not necessarily result in a worse 5-year overall survival rate [24]. The authors of that study recommended that patients who undergo subtotal resection should be monitored closely for recurrence, and salvage surgery should be considered if a recurrence occurs.

As for the role of radiation therapy or chemotherapy in CPP cases, the data remain unclear. In the current cohort, only fourteen patients with CPP had received radiotherapy, and four patients with CPP had received chemotherapy. Further research and studies are required to better comprehend the potential role of radiation therapy or chemotherapy in managing CPP cases effectively. More extensive investigations will help shed light on the significance of radiation therapy or chemotherapy as a treatment option for CPP.

### 4.2. Choroid Plexus Carcinoma

Similar to prior studies, our study showed a significantly improved OS in patients with CPC who received gross total resection [1,9,15,18,25]. Gross total resection of tumors may be challenging due to factors such as tumor location, hypervascularity, and the complexities of operating on young patients. This can increase the chances of morbidity or make surgical intervention impractical. For patients with subtotal resections, a second-look surgery is often considered, especially after chemotherapy, to potentially achieve gross total resection [4]. Unfortunately, the SEER database only records data from the initial treatment course, making it difficult to draw conclusions about relapse frequency.

Our study showed a significant improvement in OS with the use of neoadjuvant therapy. When looking at those patients who received subtotal resection alone compared to patients who received gross total resection, those that received subtotal resection had poorer OS. However, the majority of patients who received subtotal resection also received neoadjuvant therapy in the form of radiation, chemotherapy, or both. Furthermore, it is interesting to note that those that receive subtotal resection with any form of neoadjuvant treatment had no difference in OS compared to patients who received gross total resection. Thereby, these results suggest that there is a critical role that neoadjuvant therapies play in improvement in survival, especially in those patients who receive subtotal resection. We acknowledge that there might be some inherent selection bias in patients who received only subtotal resection, as their presentation might have been more aggressive in nature. This could have made it impossible for either adjuvant treatment or contraindicated adjuvant treatment. Some accounts of published cases suggest the advantage of radiation regardless of the extent of resection, while others indicate its benefit only in patients who undergo subtotal resection [9,14,15]. Due to the known neurocognitive effects of CNS irradiation, particularly in young patients commonly affected by this disease, further data are necessary to determine the benefits of adjuvant radiation [14].

Interestingly, our study shows no difference in OS with adjuvant chemotherapy in patients with CPC regardless of the extent of resection. This is one of the first SEER database studies to report data on chemotherapy use in patients with CPTs to our knowledge. The international CPT-SIOP-2000 study was the first study to standardize a treatment approach for chemotherapy use in patients with CPTs. In their study, patients with CPC, disseminated disease, or subtotally resected aCPP received six cycles of chemotherapy (etoposide, vincristine, and randomization to carboplatin or cyclophosphamide). Their results show the feasibility and effectiveness of chemotherapy in treating CPC. In addition, it highlighted the fact that CPC can be cured without irradiation in a subset of patients [26]. In our study, it was also very interesting to see that patients who received only chemotherapy also had no difference in OS when compared to patients who received gross total resection. Given that surgery is the primary treatment option for patients with CPC, there is no further literature investigating treatment with only chemotherapy in patients with CPC. Some reports suggest that patients with low structural variation in TP53 (or TP53-immunonegative) respond well to chemotherapeutic options [27]. With ongoing advancements in chemotherapy, future treatment protocols should increasingly prioritize chemotherapy as a primary treatment option for these patients.

### 4.3. Nomogram

In the field of medicine, especially in cancer research and treatment, nomograms play a crucial role as a valuable tool to assist in making treatment decisions. These predictive models combine various clinical, pathological, and molecular factors to provide estimates on a patient’s chances of survival or the risk of cancer recurrence. This information proves immensely beneficial, enabling clinicians to customize treatment plans based on each patient’s unique needs and monitor disease progression effectively over time. Nomograms are designed to handle insignificant prognostic factors by assigning appropriate weights to each factor based on its significance in predicting outcomes. In the process of developing a nomogram, statistical analyses are performed to determine the relevance and impact of each prognostic factor on the outcome of interest. Factors that are found to have little or no significant impact on the outcome are given lower weights or may even be excluded from the nomogram altogether. This ensures that only the most relevant and influential factors are incorporated into the final predictive model, improving its accuracy and clinical utility.

Nomograms represent the future of prognostic survival data modeling, gaining popularity due to their ability to simplify complex predictive survival models into a numerical point-based system for estimating event probabilities, such as overall survival (OS) [28]. These tools provide a robust and practical means for clinicians to stratify the risk of survival in patients with rare tumors like CPTs. For the purposes of this study, we were interested in developing a nomogram that considered each individual treatment modality. Although some of these factors were not found to be statistically significant based on the Cox proportional hazard models, we were more interested in how treatment modalities can be optimized for patients to maximize OS. The nomogram developed in this study is the first ever for patients with CPC. Our primary focus for this nomogram was to highlight the survival benefits for specific treatments in hopes to guide clinicians in their decision-making process for the care of these patients.

One important consideration with nomograms is that their strength relies on the amount of data used to train the model. As more data are gathered, the nomogram can be further refined and improved. As advancements in chemotherapy continue, future treatment protocols might favor chemotherapy as a primary treatment option for these patients.

### 4.4. Study Limitations

The Cox proportional hazard model, while widely used in survival analysis, has limitations, including the assumption of proportional hazards over time. Violations of this assumption can lead to biased results. Stratifying the analysis by the variable that violates the proportional hazards assumption allows for separate baseline hazard functions, accommodating differences in the hazard ratios over time. In this study, we stratify each variable to individually assess their effect on survival as one of the first steps in our analysis. However, it is still important to consider this limitation when understanding survival data.

This study has some inherent limitations, as is often the case with research based on SEER data. One notable limitation is the absence of information regarding chemotherapy, radiation, or salvage therapies, which can impact the overall analysis. Additionally, potential selection biases may have resulted in patients with more unfavorable disease characteristics being primarily treated with radiation, potentially masking any potential benefits in cases of CPC. Another limitation that is inherent to this analysis is the lack of information on the timing of treatment. While the database reports the time to treatment from diagnosis, it does not provide details about which treatment was initiated first or the specific timing between each treatment, particularly for participants who received combination therapy. This absence of timing data makes it challenging to fully understand the sequential order and intervals between treatments for these individuals. Furthermore, the lack of central pathology review raises concerns about the generalizability of the study’s findings, as this could influence the accuracy and consistency of the results. Other data on the progression of disease post-treatment, perioperative performance status, recurrence rate of tumor, and repeat surgery were not included in this database. It is important to acknowledge and consider these limitations when interpreting the outcomes of this study. Eventually, incorporating all these factors in the SEER database will lead to a more comprehensive understanding of the clinical features and overall effectiveness of the therapies.

Retrospective studies, reliant on existing data, possess inherent limitations, including selection bias, potential inaccuracies or incompleteness in data, and challenges in controlling for confounding variables. The temporal sequence of events may be ambiguous, and the lack of experimental control limits the study’s internal validity. While the SEER database is an expansive database encompassing multiple registries throughout the United States, database-specific constraints, such as coding errors or biases, further contribute to potential inaccuracies. Generalizability is often restricted, and survivorship bias may impact the estimation of survival rates. Researchers must be cautious in interpreting findings, ensuring a nuanced understanding of the retrospective study’s validity and relevance.

## 5. Conclusions

The present study is the largest SEER database analysis looking at outcomes in patients with CPTs. This study demonstrates the survival difference between patients with CPP compared to patients with CPC. It highlights some of the optimum treatment options available for the treatment of both CPP and CPC. This study also provides a better understanding of neoadjuvant therapies, such as radiation and chemotherapy, for the treatment of these rare tumors and their benefit to survival in patients. However, we still believe that further prospective studies are required to clearly define the optimal combination of treatment options available for patients with CPTs. We hope that the nomograms developed in this study can serve as a crucial tool for clinicians in guiding treatment for patients with CPTs.

## Figures and Tables

**Table 1:** Demographics, tumor characteristics, and treatment regimen of patients included in the cohort.

		CPP (n = 462)			aCPP (n = 75)			CPC (n = 142)		
Category	Subcategory	Number of Patients	Percentage	Deaths	Number of Patients	Percentage	Deaths	Number of Patients	Percentage	Deaths
Sex	Female	239	52	15	34	43	6	57	40	23
	Male	223	48	20	41	52	3	85	60	32
Age	[0–18]	213	46	4	45	57	3	113	143	44
	(18–65]	220	48	20	29	37	5	29	37	11
	>65	29	6	11	1	1	1	0	0	0
Race	Non-Hispanic White	270	58	20	48	61	6	76	54	30
	Hispanic (all races)	117	25	4	21	27	2	40	28	14
	Non-Hispanic American Indian/Alaska Native	3	1	0	0	0	0	3	2	3
	Non-Hispanic Asian or Pacific Islander	33	7	3	2	3	0	7	5	2
	Non-Hispanic Black	29	6	8	4	5	1	13	9	6
	Unknown	10	2	0	0	0	0	3	2	0
Year of diagnosis	2000–2005	59	13	7	5	6	2	45	32	24
	2006–2010	145	31	14	23	29	6	30	21	13
	2010–2015	149	32	9	21	27	1	38	27	11
	2015–2019	109	24	5	26	33	0	29	20	7
Tumor Size (cm)	<3	204	44	13	23	29	3	19	13	4
	>3.0	154	33	9	40	51	6	81	57	27
	Unknown	104	23	13	12	15	0	42	30	24
Treatment	GTR	273	59	16	46	58	5	30	20	10
	Chemo	0	0	0	0	0	0	4	3	2
	GTR + Chemo	1	0	0	1	1	1	39	26	11
	GTR + Chemo + Rad	0	0	0	0	0	0	11	7	6
	GTR + Rad	3	1	0	0	0	0	12	8	3
	Rad	6	1	4	23	29	3	1	1	1
	STR	172	37	14	2	3	0	8	5	5
	STR + Chemo	2	0	0	0	0	0	27	18	13
	STR + Chemo + Rad	1	0	1	2	3	0	6	4	2
	STR + Rad	4	1	0	0	0	0	4	3	2
	Chemo + Rad	0	0	0	1	1	0	0	0	0

Abbreviations: GTR = gross total resection; STR = subtotal resection; Chemo = chemo-therapy; Rad = radiation.

**Table 2:** Univariate cox proportional hazards analyses of overall survival.

		CPP (n = 462)			aCPP (n = 75)			CPC (n = 142)		
Category	Subcategory	HR	95% CI	*p*-Value	HR	95% CI	*p*-value	HR	95% CI	*p*-Value
Sex	Female	reference		0.3	reference		0.4	reference		0.9
	Male	1.46	[0.75–2.86]		0.52	[0.13–2.12]		1.04	[0.61–1.78]	
Age	[0–18]	reference		5.00 × 10^−15^	reference		0.01	reference		0.7
	(18–65]	5.24	[1.79–15.4]	**	2.36	[0.56–9.91]		0.87	[0.45–170]	
	>65	31.1	[9.74–99.3]	***	16.1	[1.61–160]	**	NA	NA	
Race	Non-Hispanic White	reference		1.00 × 10^−3^	reference		0.8	reference		0.02
	Hispanic (all races)	0.5	[0.17–1.47]		0.81	[0.16–4.01]		0.76	[0.40–1.44]	
	Non-Hispanic American Indian/Alaska Native	NA	NA		NA	NA		5.38	[1.60–18.1]	**
	Non-Hispanic Asian or Pacific Islander	1.28	[0.38–4.33]		NA	NA		0.81	[0.19–3.39]	
	Non-Hispanic Black	3.99	[1.76–9.07]	***	2.22	[0.27–18.5]		0.89	[0.36–2.14]	
	Unknown	NA	NA		NA	NA		NA	NA	
Year of diagnosis	2000–2005	reference		1	reference		0.06	reference		0.8
	2006–2010	1.01	[0.39–2.63]		0.59	[0.12–2.96]		0.81	[0.41–1.61]	
	2010–2015	0.9	[0.31–2.66]		0.1	[0.01–1.17]		0.69	[0.33–1.44]	
	2015–2019	0.93	[0.26–3.23]		NA	NA		0.93	[0.38–2.24]	
Tumor Size (cm)	<3	reference		0.3	reference		0.3	reference		0.2
	>3.0	0.97	[0.41–2.27]		1.49	[0.37–5.98]		1.8	[0.63–5.14]	
	Unknown	1.64	[0.76–3.56]		NA	NA		2.52	[0.87–7.29]	
Treatment	GTR	reference		5.00 × 10^−10^	reference		0.1	reference		0.4
	Chemo	NA	NA		NA	NA		1.57	[0.34–7.26]	
	GTR + Chemo	NA	NA		11.5	[1.28–103]	*	0.92	[0.39–2.21]	
	GTR + Chemo + Rad	NA	NA		NA	NA		1.66	[0.60–4.61]	
	GTR + Rad	NA	NA		NA	NA		0.73	[0.20–2.66]	
	Rad	15.6	[5.15–47.7]	***	NA	NA		2.47	[0.31–19.5]	
	STR	1.57	[0.76–3.23]		1.59	[0.38–6.71]		3.26	[1.11–9.59]	*
	STR + Chemo	NA	NA		NA	NA		1.68	[0.73–3.86]	
	STR + Chemo + Rad	28.6	[3.70–220]	***	NA	NA		1.11	[0.24–5.08]	
	STR + Rad	NA	NA		NA	NA		1.81	[0.39–8.32]	
	Chemo + Rad	NA	NA		NA	NA		NA	NA	

(‘***’ represents *p* < 0.001; ‘**’ represents *p* < 0.01; ‘*’ represents *p* < 0.05) (Abbreviations: HR = hazard ratio; CI = confidence interval; GTR = gross total resection; STR = subtotal resection; Chemo = chemotherapy; Rad = radiation).

## Data Availability

All analysis was performed using in-house code, which can be provided at any time with an email request to the corresponding author. Data are publicly available.
